# Tcf7l2 Is Required for Left-Right Asymmetric Differentiation of Habenular Neurons

**DOI:** 10.1016/j.cub.2014.08.006

**Published:** 2014-10-06

**Authors:** Ulrike Hüsken, Heather L. Stickney, Gaia Gestri, Isaac H. Bianco, Ana Faro, Rodrigo M. Young, Myriam Roussigne, Thomas A. Hawkins, Carlo A. Beretta, Irena Brinkmann, Alessio Paolini, Raquel Jacinto, Shahad Albadri, Elena Dreosti, Matina Tsalavouta, Quenten Schwarz, Florencia Cavodeassi, Anukampa K. Barth, Lu Wen, Bo Zhang, Patrick Blader, Emre Yaksi, Lucia Poggi, Mihaela Zigman, Shuo Lin, Stephen W. Wilson, Matthias Carl

**Affiliations:** 1 Department of Cell and Molecular Biology, Medical Faculty Mannheim, Heidelberg University, Ludolf-Krehl-Strasse 13-17, 68167 Mannheim, Germany; 2 Department of Cell and Developmental Biology, University College London, Gower Street, London WC1E 6BT, UK; 3 Centre de Biologie du Développement (CDB), UPS, Université de Toulouse, 118 Route de Narbonne, 31062, France; 4 CNRS, CDB UMR 5547, 31062 Toulouse, France; 5 Centre for Organismal Studies, Heidelberg University, 69120 Heidelberg, Germany; 6 Neuroelectronics Research Flanders, 3001 Leuven, Belgium; 7 Institute of Ophthalmology, University College London, London EC1V 9EL, UK; 8 Key Laboratory of Cell Proliferation and Differentiation of the Ministry of Education, College of Life Sciences, Peking University, Beijing 100871, China; 9 Vlaams Instituut voor Biotechnologie, 3001 Leuven, Belgium; 10 KU Leuven, 3001 Leuven, Belgium; 11 Department of Molecular Evolution and Genomics, Centre for Organismal Studies (COS), Heidelberg University, Im Neuenheimer Feld 329, 69120 Heidelberg, Germany; 12 Department of Molecular, Cell, and Developmental Biology, University of California, Los Angeles, 621 Charles E. Young Drive, Los Angeles, CA 90095, USA

## Abstract

**Background:**

Although left-right asymmetries are common features of nervous systems, their developmental bases are largely unknown. In the zebrafish epithalamus, dorsal habenular neurons adopt medial (dHbm) and lateral (dHbl) subnuclear character at very different frequencies on the left and right sides. The left-sided parapineal promotes the elaboration of dHbl character in the left habenula, albeit by an unknown mechanism. Likewise, the genetic pathways acting within habenular neurons to control their asymmetric differentiated character are unknown.

**Results:**

In a forward genetic screen for mutations that result in loss of habenular asymmetry, we identified two mutant alleles of *tcf7l2*, a gene that encodes a transcriptional regulator of Wnt signaling. In *tcf7l2* mutants, most neurons on both sides differentiate with dHbl identity. Consequently, the habenulae develop symmetrically, with both sides adopting a pronounced leftward character. Tcf7l2 acts cell automously in nascent equipotential neurons, and on the right side, it promotes dHbm and suppresses dHbl differentiation. On the left, the parapineal prevents this Tcf7l2-dependent process, thereby promoting dHbl differentiation.

**Conclusions:**

Tcf7l2 is essential for lateralized fate selection by habenular neurons that can differentiate along two alternative pathways, thereby leading to major neural circuit asymmetries.

## Highlights

• Zebrafish with mutations in *tcf7l2* lose left-right asymmetries in habenular neurons
• Tcf7l2 is expressed in both left and right-sided habenular neurons
• Tcf7l2 enables neurons to respond to signals that differ between left and right

## Teaser

Although left-right asymmetries are common features of nervous systems, their developmental bases are largely unknown. This study in zebrafish by Hüsken et al. reveals that Tcf7l2 is essential for lateralized fate selection by neurons that can differentiate along two alternative pathways, thereby leading to major neural circuit asymmetries.

## Introduction

Left-right (LR) asymmetries in the nervous system are believed to contribute to a wide variety of cognitive processes and behaviors [1, 2]. However, there are only very few cases for which it is known how the asymmetries arise and how they are represented at the level of individual neurons, and there are fewer still for which functional roles for the asymmetries have been assigned [1].

The most well-conserved brain asymmetries in vertebrates are within the diencephalic epithalamus, which contains the pineal complex and the adjacent habenular nuclei [3, 4]. The habenular nuclei are a central component of a series of limbic system pathways that convey information from various forebrain nuclei to dopaminergic and serotonergic output pathways [4, 5, 6]. The habenular nuclei are implicated in providing negative reward signals and in mediating innate and learned responses to aversive stimuli and various other behaviors, and their pathophysiology is linked to neurological conditions including depression and schizophrenia [6, 7]. The habenulae show asymmetries in all vertebrate classes, although the nature and extent of the asymmetries varies considerably [3].

Progress has been made in elucidating the mechanistic and genetic basis of the stereotyped laterality (handedness) of epithalamic asymmetries. In zebrafish, the parapineal nucleus is located on the left side of the brain and exclusively innervates the left habenula [8, 9]. Parapineal precursors initially occupy a symmetric position at the dorsal midline of the brain but subsequently migrate to the left [9]. The left-sided position of the parapineal is dependent upon local asymmetric left-sided Nodal signaling, which provides directionality to the Fgf-dependent migration of parapineal precursors from their initial midline location [10, 11]. The asymmetric activation of Nodal signaling in the brain is dependent upon left-sided Nodal pathway activation by the ligand Southpaw in the lateral plate mesoderm (LPM) [8, 12]. One current model postulates that Wnt signaling inhibits Nodal pathway activation on both sides of the brain, potentially through suppression of Six3 activity, and that this repression is alleviated on the left through the action of Southpaw [12, 13, 14, 15].

Habenular asymmetry is manifested as a left-right (LR) difference in the proportion of dorsal habenula (dHb) projection neurons that elaborate medial (dHbm) versus lateral (dHbl) subnuclear character [16, 17, 18]. On the left, the dHbl is larger, whereas on the right, it is the dHbm subnucleus that is larger. These subnuclei project to different regions of the target interpeduncular nucleus (IPN) in the ventral midbrain. Therefore, habenular asymmetry results in laterotopic efferent connectivity wherein the majority of left habenular neurons (dHbl identity) innervate the dorsal IPN, whereas most neurons on the right (dHbm identity) connect with the ventral IPN [16, 17, 18]. Dorsal and ventral IPN neurons subsequently project to different regions [19], suggesting that predominantly left- and right-sided habenular activity may have different behavioral consequences. Indeed, habenular lateralization is required for dHb neurons to respond appropriately to olfactory and visual stimuli [20].

The molecular mechanisms by which the dHb acquires LR asymmetry are not well understood. Ablation experiments indicate that parapineal cells communicate with habenular cells on the left and that this communication is required for neurons to elaborate dHbl character [9, 16, 18, 21]. The nature of the communication between parapineal and habenular cells is unknown. Notch signaling influences the timing of production of dHb neurons, with those born early having a greater likelihood to differentiate with dHbl character and those born later with dHbm character [22]. Influenced by left-sided Nodal signaling, neuronal production occurs earlier on the left than on the right, and this difference could consequently contribute to the difference in proportions of dHbl and dHbm neurons on the left and right [22, 23]. It is unclear whether the parapineal has any influence on the timing of habenular neurogenesis or promotes dHbl character through a different mechanism. It is also unknown how habenular precursors and/or neurons respond to lateralized intrinsic and extrinsic factors to elaborate different programs of differentiation.

In order to elucidate the genetic basis by which dHb neurons acquire lateralized dHbl/dHbm identity, we undertook a forward genetic screen to identify mutations that lead to the loss of habenular asymmetry. We isolated two mutant alleles of *tcf7l2*, a gene that encodes a transcriptional effector of Wnt/β-catenin signaling [24], that can also function independently of β-catenin in some contexts [25]. These two and another allele [26] all show a loss of dHb asymmetry, whereas lateralized Nodal pathway gene expression and visceral organ positioning are unaffected. Tcf7l2 is expressed in dHb neurons on both left and right as they initiate differentiation. On the right, Tcf7l2 cell autonomously prevents adoption of dHbl identity, and in the absence of Tcf7l2 function, most neurons on both the left and right differentiate with dHbl character. In mutants in which Wnt signaling is enhanced [13] and in embryos in which the parapineal is ablated [9, 16, 18, 21], most neurons on both sides adopt dHbm identity, and we find that these phenotypes require Tcf7l2. Our results suggest that Tcf7l2 enables nascent dHb neurons to respond appropriately to signals in the environment into which they are born in a LR-specific manner, such that neurons on the left and right sides of the brain follow distinct programs of differentiation.

## Results

### *tcf7l2* Is Required for the Establishment of Habenular Asymmetry

**Figure 1:**
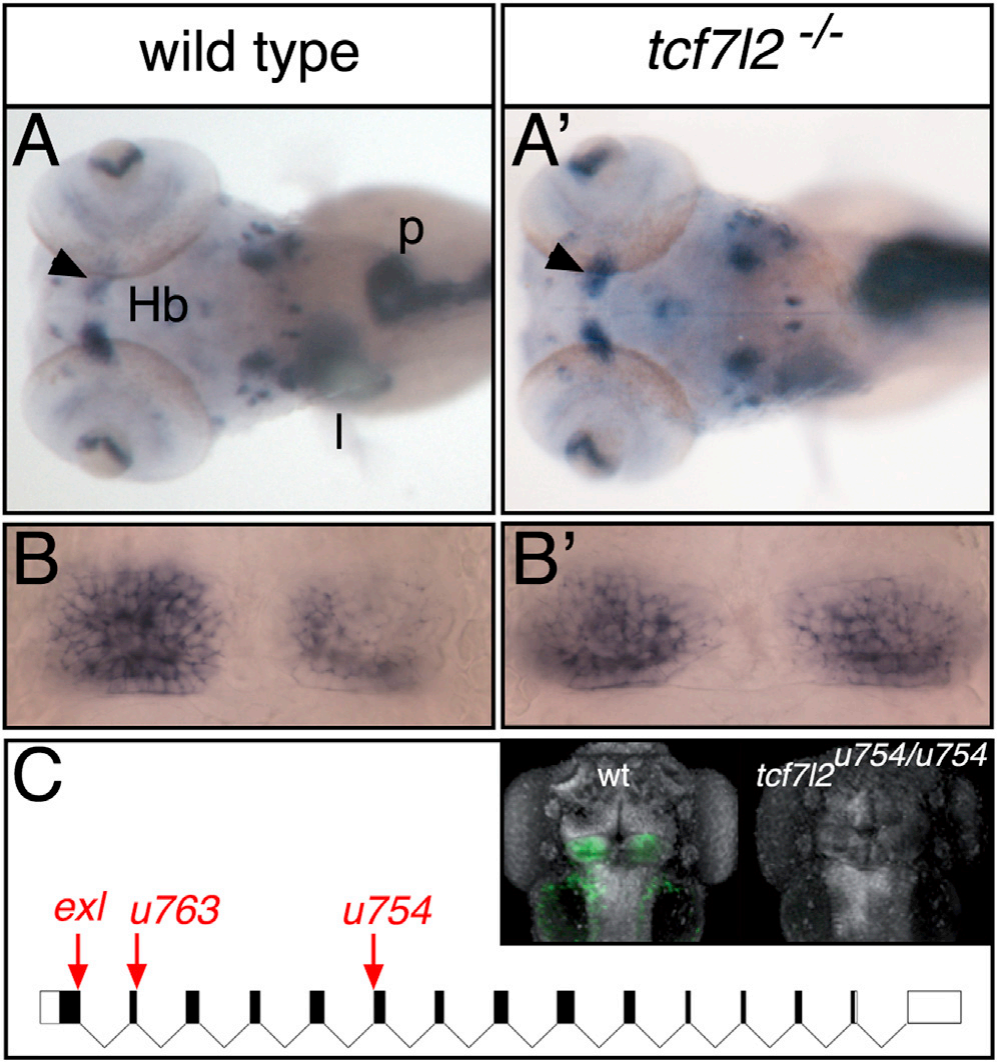
*tcf7l2* Is Required for the Establishment of Habenular Asymmetry (A and A′) Dorsal views of 4 dpf (days postfertilization) embryos, with anterior to the left, labeled with a cocktail of markers that reveal habenular asymmetry (*kctd12.1*) and position of liver (*sepB*) and pancreas (*trypsin*). The arrowheads indicate normal (A) and increased (A′) *kctd12.1* expression in the right habenula. (B and B′) Dorsal views of the habenulae with anterior to the top showing symmetric *kctd12.1* expression in a wild-type fry (B) and a *tcf7l2* mutant (B′). (C) Diagram of intron/exon structure for *tcf7l2* showing the *tcf7l2*^exl^ [26], *tcf7l2*^u763^, and *tcf7l2*^u754^ alleles. Arrows point to mutation sites. The inset shows dorsal views, with anterior to the top, of 4 dpf wild-type (wt) and *tcf7l2*^u754/u754^ mutant embryos stained for Tcf7l2 (green) and Sytox orange (gray). Hb, habenula; l, liver; p, pancreas. See also Figure S1.

To identify genes involved in the determination of brain asymmetries, we screened the progeny of F2 families carrying *N*-ethyl-*N*-nitrosourea-induced mutations with a cocktail of markers that revealed habenular asymmetry along with the position of the liver and pancreas (Figure 1A). This approach allowed us to distinguish between mutations causing asymmetry defects in both body and brain and those affecting only brain asymmetry.

We identified two noncomplementing mutations, *u754* and *u763*, that lead to symmetric habenular expression of *left-over/kctd12.1* [21] in about 25% of embryos from sibling pair matings (phenotypes present in 21.8% of 541 and 29.9% of 127 embryos from *u754* and *u763* carriers, respectively; Figures 1A–1B′). Mutant fish are morphologically indistinguishable from wild-types, and asymmetry and laterality of the liver and pancreas are unaffected (Figures 1A and 1A′). Consistent with this, left-sided expression of *southpaw* (*spw*) [12] in the LPM and and the Nodal target *pitx2* in both LPM and epithalamus are unaffected (Figures S2A–S2B′ and Table S1 available online; some data from analysis of the *exl* allele [26] are described below). Consequently, *u754* and *u763* are likely to be mutations in the same gene that lead to loss of habenular asymmetry despite normal left-sided Nodal signaling.

**Figure 2:**
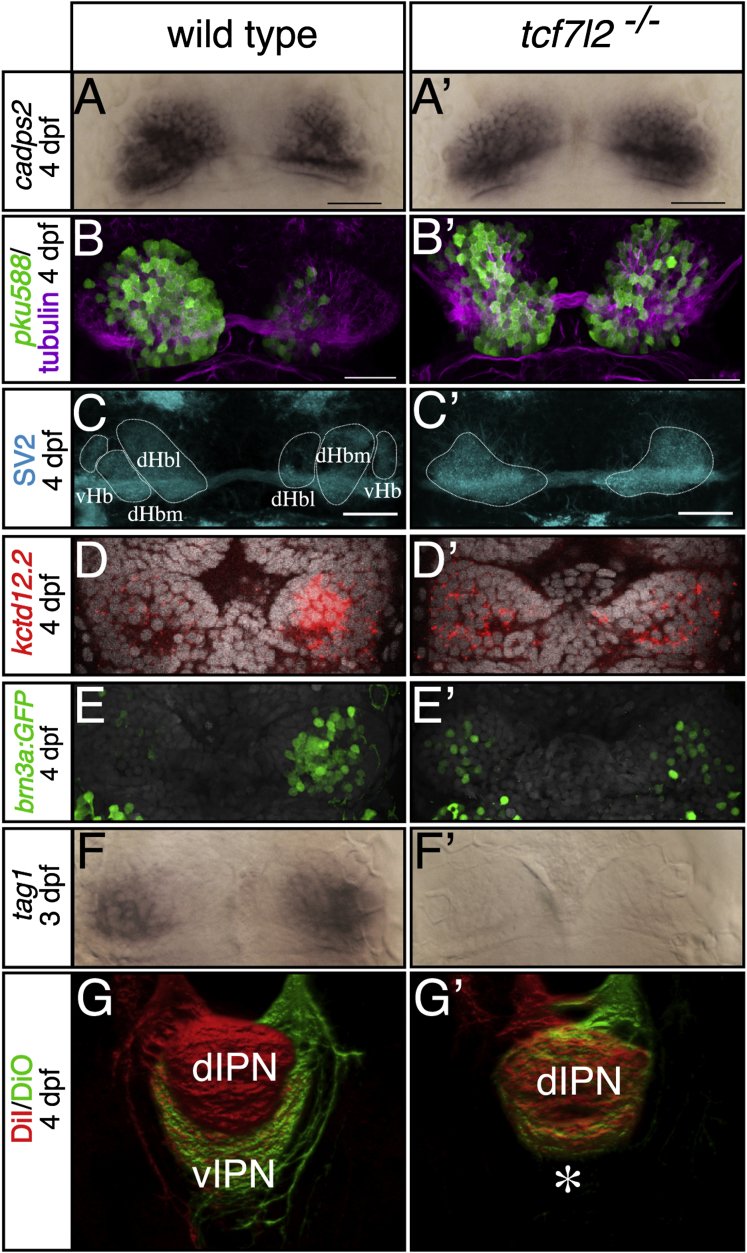
*tcf7l2* Mutants Show LR Symmetric Patterns of Epithalamic Neuron Differentiation (A–F′) Dorsal views, with anterior to the top, of the habenulae of embryos labeled with markers at stages shown to the left. In (D–E′), nuclei (gray) are labeled with Topro3 and Sytox orange, respectively. All markers are symmetric in *tcf7l2* mutants. Scale bars in (A)–(C′) represent 25 μm. (G and G′) Dorsal views of 3D reconstructed confocal images of lipophilic-dye-labeled left (red) and right (green) habenular axons terminating in the IPN. The asterisk highlights the lack of axon terminals normally innervating the ventral IPN. dHbl and dHbm, lateral and medial habenular subnucleus, respectively; dIPN and vIPN, dorsal and ventral interpeduncular nucleus, respectively; vHb, ventral habenular subnucleus. See also Figures S2 and S3 and Table S1.

Genetic mapping using simple sequence length polymorphism (SSLP) markers localized the *u754* mutation to a 9.2 Mb interval on LG12 that contains *tcf7l2* (Figure S1A), a gene encoding a Tcf family transcription factor. Sequencing of genomic DNA from mutants revealed a splice donor site lesion at exon II of the *tcf7l2* gene in *u763* and a splice acceptor site lesion at exon VI in *u754* (Figures 1C and S1B), suggesting that disrupted Tcf7l2 function is responsible for the observed asymmetry phenotype. *u754* fails to complement the *tcf7l2*^*exl*^ allele [26], which harbors a mutation in the splice acceptor site of exon I (Figure 1C) and which we find also leads to development of symmetric habenulae (Figures 2 and S2). Tcf7l2 immunoreactivity is absent in *u754* mutants, suggesting that it is a null allele (Figure 1C inset; see the Experimental Procedures). Consequently, we conclude that Tcf7l2 function is required for habenular neurons to acquire LR asymmetry.

### dHb Neurons on Both Sides Differentiate with Left-Typical Character in *tcf7l2* Mutants

**Figure 3:**
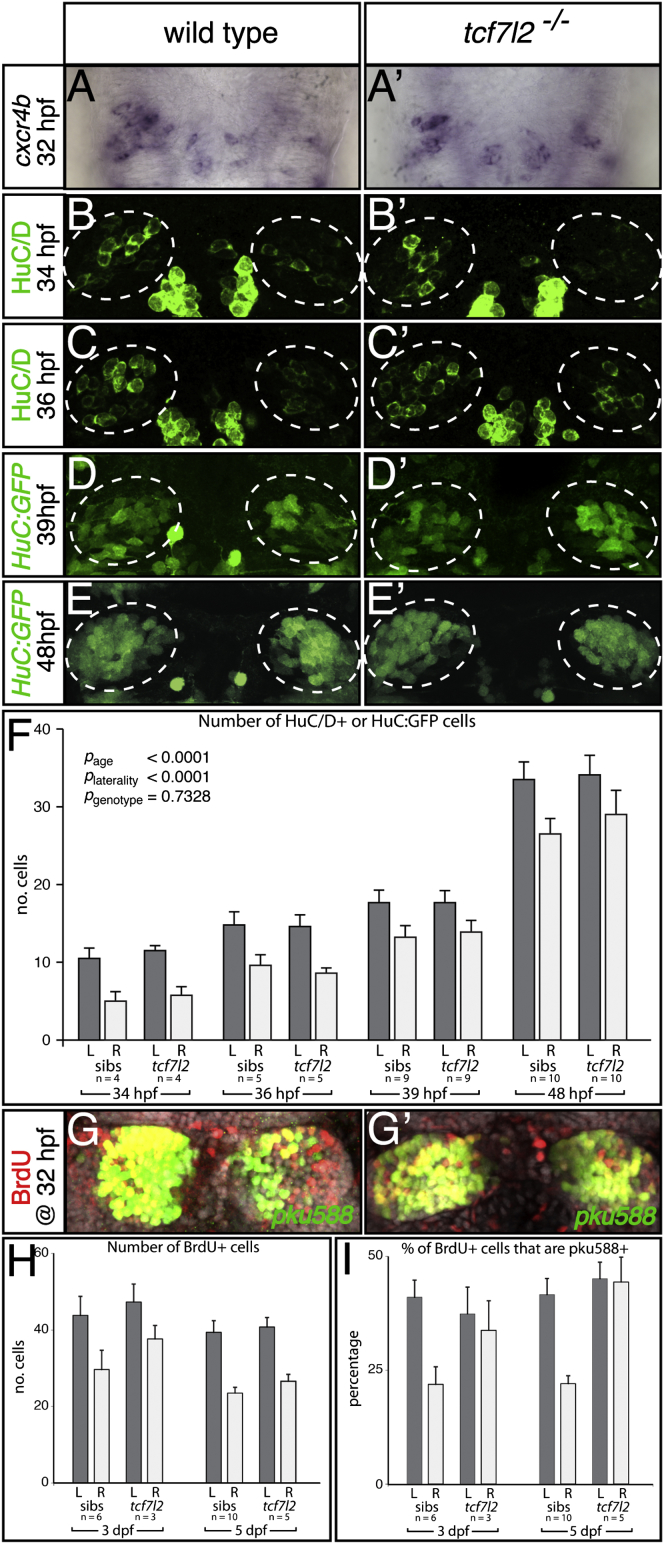
Habenular Neurogenesis Is Not Overtly Affected in *tcf7l2* Mutants (A–E′, G, and G′) Dorsal views of the epithalamus, with anterior to the top. (A and A′) The numbers and LR asymmetry of *cxcr4b*-expressing dHb neuronal precursors is not overtly affected in the *tcf7l2* mutant. (B–E′) HuC/D (B–C′) and transgenic *HuC:GFP* (D–E′) expression in differentiating habenular neurons in wild-type fry and *tcf7l2* mutants. The dotted circles highlight the dHb. (F) Numbers of HuC/D^+^ and *HuC:GFP*^*+*^ neurons are not significantly different between wild-type embryos and *tcf7l2* mutants at 34, 36, 39, and 48 hpf. Error bars indicate the SEM. (G and G′) BrdU (red)-labeled, 5 dpf dHb neurons expressing the *pku588* transgene (green) in a wild-type (G) and a *tcf7l2* mutant (G′). (H and I) Graphs representing the number of BrdU-labeled cells (H) and the percentage of BrdU-labeled cells coexpressing the *pku588* transgene (I) in wild-type embryos and *tcf7l2* mutants. Error bars indicate the SD. See also Table S2.

Phenotypic analysis of genotyped embryos confirmed a fully penetrant symmetric habenular phenotype in the absence of functional Tcf7l2 (Figure 2 and Table S1; true for *exl*, *u754* and *u763* alleles). *cadps2* (*cpd2*) is broadly expressed in both the left and right dHb [16], and expression was similarly robust and symmetric in *tcf7l2* mutants at 4 dpf, suggesting that there is no overt difference in the size of the dHb (Figures 2A and 2A′). Similarly, there is no significant difference in the overall numbers of *HuC:GFP* [27] expressing habenular neurons in the mutants (see below; Figures 3D–3F and Table S2). The marker originally used to identify mutant phenotypes, *kctd12.1*, is most strongly expressed in mature projection neurons of the dHbl [17, 21, 22], and so we assessed other markers of dHb neurons to determine their character in *tcf7l2* mutants. The *gata2a:eGFP*^*pku588*^ enhancer trap transgene (hereafter, *pku588* transgene) is expressed predominantly in dHbl neurons (Figure S3), and, mirroring the changes in *kctd12.1* expression, is robustly upregulated in right-sided neurons in *tcf7l2* mutants (Figures 2B and 2B′; n = 27/28). Reflecting the symmetry in neurons, habenular neuropil is symmetric (Figures 2B and 2B′; n = 10/12) and is not divided into subnuclear domains (Figures 2C and 2C′ and Movies S1 and S2). Markers that show greater expression in right-sided dHb neurons in wild-type embryos showed reduced, dispersed, or absent expression in both the left and right dHb in *tcf7l2* mutants. At 4 dpf, *right-on/kctd12.2* [21] expression is reduced and is more diffuse and symmetric in *tcf7l2* mutants than in wild-type fry (Figures 2D and 2D′; n = 17/17). Neurons restricted to the medial subnucleus of the dHb express the *Tg(hsp70-brn3a:GFP)*^*rw0110b*^ transgene [22] and so are more abundant on the right in wild-type embryos. In most mutants (n = 9/15), transgene expression is reduced on the right, and the few labeled neurons are dispersed in both the left and right dHb (Figures 2E and 2E′). *tag1* (n = 7/7), *dexter/kctd8* (n = 34/34), and *slc18a3b* (n = 15/15) expression is normally stronger in the right dHb [13, 21, 28], and expression is reduced or absent in all mutants (Figures 2F, 2F′, and S2C–S2D′ and Table S1). More dHb neurons on the left respond to a nonlateralized light pulse than on the right in wild-type fry [20], and in *tcf7l2* mutants this asymmetry is lost, with neurons in both habenulae showing robust light responses (Figures S2E and S2E′).

dHb efferent axons project to the IPN in the ventral midbrain, with axon terminals segregating laterotopically within the IPN dependent on their subnuclear origin [17, 18, 21]. Axons from dHbl neurons (more frequent on the left) target the dorsal IPN, whereas axons from dHbm neurons (more frequent on the right) innervate the ventral IPN. Therefore, axonal targeting is a good readout of dHb neuron subtypes. Habenular efferent axons in *tcf7l2* mutants fail to laterotopically segregate and only target the dorsal IPN (Figures 2G and 2G′; n = 6/6), suggesting that most if not all of the projection neurons on both left and right are of the left-typical subtype [18]. Thus, with respect to axonal targeting, molecular markers and functional response properties, the habenulae display an exaggerated “left” phenotype on both sides of the brain in *tcf7l2* mutants. Together, our results indicate that there are similar numbers of habenular neurons in the absence of functional Tcf7l2, but most neurons on both left and right acquire dHbl character, and the habenulae are therefore symmetric. These results suggest that Tcf7l2 influences the subnuclear character of dHb neurons rather than their production.

### LR Asymmetry in the Timing of dHb Neurogenesis Is Not Overtly Affected in *tcf7l2* Mutants, but the Probability of Neurons Acquiring dHbl Identity Is Increased

Production of dHb neurons peaks earlier on the left than on the right, and neurons born early are more likely to adopt dHbl identity [22]. Consequently, if dHb neurons are born early on both left and right in *tcf7l2* mutants, then this could provide an explanation for the increased presence of dHbl neurons in the right dHb. To address whether this is likely to be the case, we examined the timing of production of neurons in *tcf7l2* mutants.

*cxcr4b* is expressed in dHb neuronal precursors around the time at which they generate postmitotic neurons [23], and the timing, asymmetry, and extent of expression are not affected in 32 hpf (hours postfertilization) mutants (Figures 3A and 3A′ and Tables S1 and S2). The first HuC/D-positive dHb neurons appear at about 32–33 hpf, with a slight asymmetry, such that neurons appear on the left a few hours before the right [22, 23]. We saw no significant effect of the *tcf7l2* mutation on the asymmetric production of HuC/D-positive or *HuC:GFP*-transgene-positive neurons, as assessed by counting of cells at 34, 36, 39, and 48 hpf (p_genotype_ = 0.73, p_laterality_ < 0.001, p_age_ < 0.001, no significant first-order interactions, ANOVA; Figures 3B–3F and Table S2). BrdU pulse labeling of proliferative cells at 32 hpf revealed no significant difference between wild-type fry and *tcf7l2* mutants in the number of labeled dHb cells when analyzed at 3 or 5 dpf (Figures 3G–3H and Table S2; p_genotype_ = 0.29, ANOVA). Both wild-type siblings and *tcf7l2* mutants displayed the LR asymmetry in BrdU labeling, as previously observed [22] (Figure 3H; p_laterality_ < 0.001, ANOVA). All together, these results suggest that precocious generation of neurons on the right is not the explanation for why dHb neurons on both left and right adopt left-sided character in *tcf7l2* mutants.

Although the early production of dHb neurons appeared to be unaffected in *tcf7l2* mutants, the probability that such neurons adopted a dHbl identity was significantly changed as assessed by the percentage of BrdU^+^ cells that later expressed the *pku588* transgene (Figure 3I). In wild-type embryos, a significantly lower proportion BrdU^+^ cells expressed the *pku588* transgene in the right dHb when compared with the left (p_left versus right_ < 0.05, ANOVA with Tukey-Kramer post test, correcting for effects due to age, which were not significant). Consequently, neurons born at the same time on the left and right do not have the same probability of acquiring dHbl versus dHbm fate. However, in *tcf7l2* mutants, this asymmetry in the fate of BrdU^+^ cells was lost, and there was no significant difference in the percentage of BrdU^+^ neurons expressing the *pku588* transgene in the right versus left dHb (p_left versus right_ > 0.05). In mutants, right dHb BrdU^+^ neurons expressed the *pku588* transgene at significantly higher frequency compared to wild-type right dHb neurons (p_*tcf7l2−/−*_ _versus wild-type_ < 0.05).

These results suggest that in *tcf7l2* mutants, the early asymmetry in production of dHb neurons is not affected, but the probability that a neuron born on the right adopts a dHbl identity is increased to the same level as on the left.

### Tcf7l2 Is Expressed in Both Left- and Right-Sided dHb Neurons

**Figure 4:**
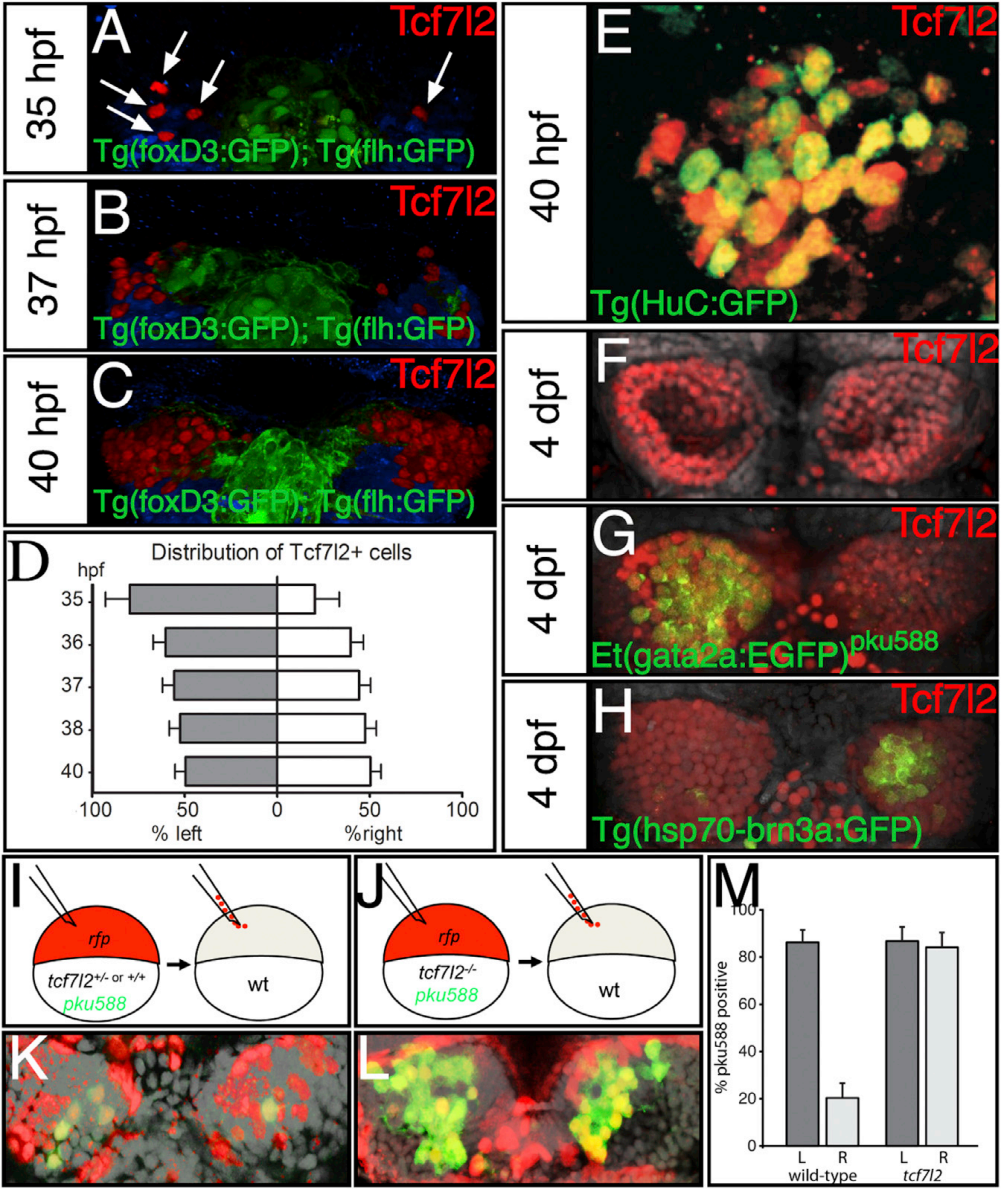
Tcf7l2 Is Expressed in Both Left- and Right-Sided dHbl and dHbm Neurons and Functions Cell/Lineage Autonomously (A–C, E–H, K, and L) Dorsal views with anterior to the top of the epithalamus at ages indicated and at 54 hpf (K and L). (A–D) Numbers of Tcf7l2 expressing cells (arrows in A) in Tg(foxD3:GFP); Tg(flh:eGFP) transgenic embryos are asymmetrically distributed in 35 and 37 hpf embryos (A and B) but are symmetric by 40 hpf (C and graph in D). Tcf7l2 expressing cells ventral to the habenulae are pseudocolored in blue. (E) High magnification of the left habenula stained for Tcf7l2 (red) and HuC:GFP (green). (F–H) Tcf7l2 expression (red) in wild-type (F), Et(gata2a:EGFP)^pku588^ (G), and Tg(hsp70-brn3a:GFP) (H) embryos colabeled with Sytox orange (gray nuclei). (I and J) Schematics of the experiment. RFP^+^ wild-type (I) or *tcf7l2* mutant (J) cells carrying the *pku588* transgene were transplanted into nontransgenic hosts in a region of the blastula from which cells later contribute to the habenulae. (K and L) Examples of transplanted control Et(gata2a:EGFP)^pku588^ × *tcf7I2*^*+/−*^ or *tcf7I2*^*+/+*^ (K) and Et(gata2a:EGFP)^pku588^ × *tcf7I2*^*−/−*^ (L) cells (donor cells are red) in wild-type habenulae (nuclei labeled gray with DAPI). (M) dHb neurons from mutant donors expressing the *pku588* transgene when incorporated into either the left or right habenula. Error bars represent the SD of the cell proportions. See also Figure S4 and Table S2.

*tcf7l2* is broadly expressed in the diencephalon ventral to the habenulae from early stages [29]; Figure S4) and, subsequent to this, in dHb neurons on both left and right sides of the brain. Mirroring the spatiotemporal pattern of neuron generation, Tcf7l2^+^ immunoreactivity is initially observed from about 35 hpf, with more neurons labeled on the left than right (Figures 4A, 4B, and 4D and Table S2). This asymmetry is gradually lost at later stages (Figures 4C and 4D and Table S2). By 40 hpf, most Tcf7l2^+^ dHb cells coexpress the postmitotic neuronal marker *HuC:GFP* (Figure 4E and Table S2). They do not colocalize with mitotic cells labeled with anti-phospho histone H3 (n = 23; data not shown). At later stages, most dHb neurons express Tcf7l2 irrespective of whether they express markers typical of dHbl or dHbm identity (Figures 4F–4H). These results indicate that the *tcf7l2* mutant phenotype could arise indirectly from a requirement for Tcf7l2 function in the diencephalon prior to production of habenular neurons or from a function within the neurons themselves.

### Tcf7l2 Functions Cell Autonomously to Prevent Expression of dHbl Character

To discriminate between these two scenarios, we transplanted cells from *tcf7l2* mutant and sibling embryos carrying the *pku588* transgene into wild-type, nontransgenic hosts and assessed whether donor-derived neurons expressed the transgene marker of dHbl neurons within a wild-type environment (Figures 4I and 4J). As expected, wild-type donor-derived neurons expressed the *pku588* transgene at high frequency in the left dHb but much less often in the right dHb of wild-type hosts (86.3% ± 5.1% express *pku588* on the left, n = 95 cells, versus 20.3% ± 6.3% on the right, n = 64 cells in ten embryos; Figures 4K and 4M). By contrast, *tcf7l2* mutant-derived dHb neurons did not display this laterality dependance and expressed the *pku588* transgene at high frequency whether on the left or on the right (86.8% ± 6.1% on the left, n = 68 cells, versus 84.1% ± 6.3% on the right, n = 63 cells in four embryos; Figures 4L and 4M). We fitted a logistical regression model to our data, which revealed a significant interaction between genotype and laterality (p < 0.001), reflecting the increase in the probability of neurons to express the *pku588* transgene when they both carry the *tcf7l2* mutation and are located on the right. In summary, this experiment demonstrated that the wild-type right-sided dHb environment is unable to prevent the adoption of left-sided character by *tcf7l2* mutant dHb neurons. The simplest explanation is that Tcf7l2 normally functions within most right-sided dHb neurons to prevent elaboration of typically left-sided character.

### The Symmetric dHb Phenotype Is Epistatic to the Effects of Parapineal Ablation

The parapineal is required for left-sided dHb neurons to elaborate dHbl subnuclear identity [9, 16, 18]. Consequently, parapineal ablation in wild-type embryos leads to the development of largely symmetric habenulae of right-sided character. It is not known how the parapineal influences the character of dHb neurons. One potential explanation for the *tcf7l2* mutant phenotype involves a requirement of Tcf7l2 function for parapineal development, migration or signaling such that parapineal signals aberrantly act upon both left and right dHb to promote left-sided character on both sides.

**Figure 5:**
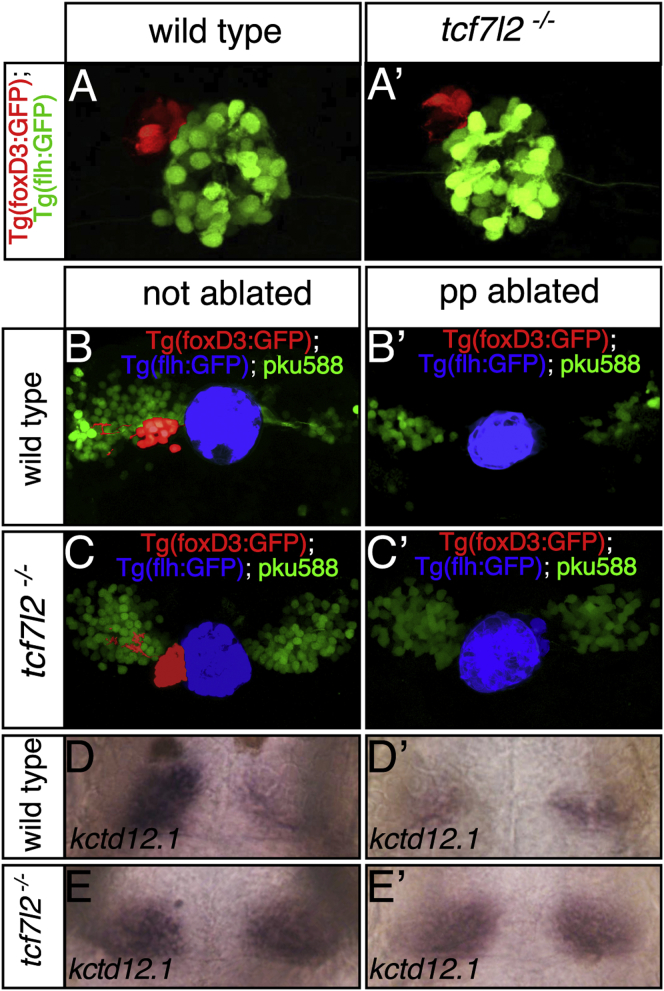
The Symmetric dHb Phenotype of *tcf7l2* Mutants Is Epistatic to Parapineal Ablation Dorsal views focused on the epithalamus of 2.5 dpf (A and A′) and 4 dpf (B–E′) embryos with anterior to the top. (A and A′) The parapineal (pseudocolored red) is on the left in both wild-type and *tcf7l2* mutant embryos. (B–E′) In Et(gata2a:EGFP)^pku588^; Tg(foxD3:GFP); Tg(flh:eGFP) triple-transgenic normal and parapineal-ablated embryos, dHbl neurons (green) and the pineal complex are labeled. The pineal is pseudocolored in blue and the parapineal and its projections in red (B–C′). Embryos as in (B)–(C′) were labeled for *kctd12.1* subsequent to live imaging (D–E′).

Despite dHb neurons on left and right acquiring symmetric, mostly left-sided character, there is only a single parapineal nucleus, positioned, as in wild-type fry, on the left in *tcf7l2* mutants (Figures 5A and 5A′ and Table S1; n = 19). Consequently, formation and migration of the parapineal does not require Tcf7l2 function.

An alternative possibility is that although the parapineal is unilateral, signals from it influence the differentiation of dHb neurons on both left and right sides in *tcf7l2* mutants. To address this possibility, we performed multiphoton laser-induced ablations of parapineal precursors prior to their migration and signaling to the habenula. Parapineal ablation in wild-type embryos led to the expected decrease of *pku588* trangene expression (n = 12/12) and *kctd12.1* (n = 24/24) expression on the left side (Figures 5B, 5B′, 5D and 5D′). However, it had no overt effect on the expression of these dHbl markers on either the left or right side in *tcf7l2* mutants (Figures 5C, 5C′, 5E, and 5E′; n = 8/8 [*pku588* transgene] and n = 21/21 [*kctd12.1*]). These data demonstrate that the symmetric dHbl phenotype of *tcf7l2* mutants is epistatic to the effects of parapineal ablation. They also indicate that the abrogation of functional Tcf7l2 has similar phenotypic consequences on both the left and right sides (parapineal independent elaboration of dHbl character), although the phenotype is only obvious on the right.

### Tcf7l2 Functions Downstream of Axin1 to Suppress dHbl and Promote dHbm Character

**Figure 6:**
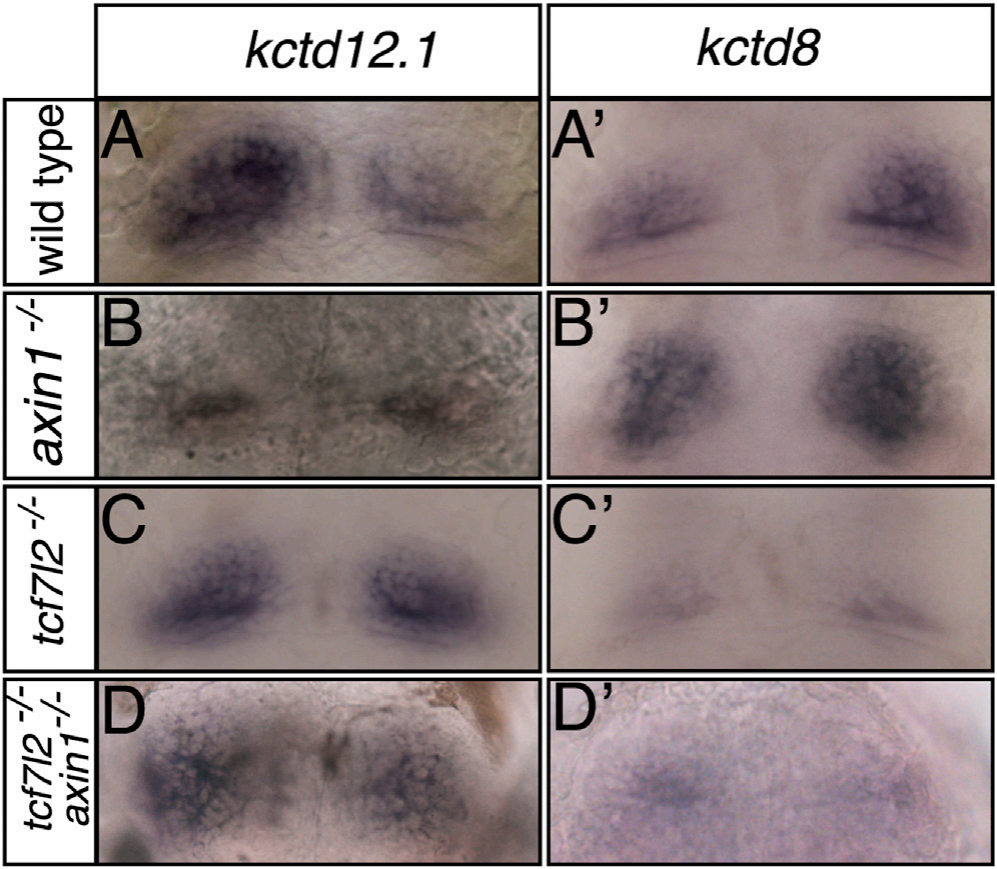
The dHb Phenotype of *tcf7l2* Mutants Is Epistatic to the Effects of a Mutation in *axin1* that Enhances Wnt Signaling Dorsal views of the habenulae of 4 dpf embryos of genotype shown to the left labeled with markers shown above columns (A–D′). The enhanced expression of *kctd12.1* and reduced expression of *kctd8* in *tcf7l2* mutants is observed irrespective of the presence (C and C′) or absence (D and D′) of functional Axin1. See also Table S3.

Tcf family members usually function as transcriptional activators or repressors of the Wnt/β-catenin pathway. In the absence of active signaling, Tcfs recruit proteins such as Groucho and Hdac to repress target genes, whereas in the presence of active signaling, β-catenin binds to Tcfs, displacing repressor components and activating target gene expression [24]. Axin1 is a component of a scaffolding complex that limits Wnt signaling by targeting β-catenin for degradation, and *axin1* (*masterblind*) mutant zebrafish embryos consequently show enhanced Wnt signaling in the brain [30, 31]. To address whether Tcf7l2 might function in a canonical Wnt/Axin1/β-catenin signaling cascade during establishment of habenular asymmetry, we addressed the epistatic relationship between mutations in *axin1* and *tcf7l2*. *Axin1* mutants have a complex epithalamic laterality phenotype, one aspect of which is that the dHb develop double-right-sided character [13, 15] (Figures 6A–6B′). If this phenotype depends upon Tcf7l2 functioning downstream of Axin1, then the double-right phenotype should be suppressed in absence of Tcf7l2 function. Indeed, *axin1/tcf7l2* double mutants show symmetric habenulae of left-sided character indistinguishable from *tcf7l2* single-mutant habenulae, as judged by the expression of *kctd12.1* and *kctd8* (Figures 6C–6D′ and Table S3). Consequently, the activation of Wnt signaling in *axin1* mutants only elicits changes in dHb subnuclear character in the presence of Tcf7l2 function. These data are consistent with both genes acting in the same pathway to establish dHb asymmetry with Axin1 acting upstream of Tcf7l2. Furthermore, as Axin1 inhibits Wnt signaling, the Tcf7l2-dependent expansion of dHbm and loss of dHbl markers in *axin1* mutants are most likely dependent upon Tcf7l2 activating Wnt signaling.

### Inhibition of Wnt Signaling during the Period of Neuronal Production Leads to Expansion of dHbl Markers in Right-Sided dHb Neurons

**Figure 7:**
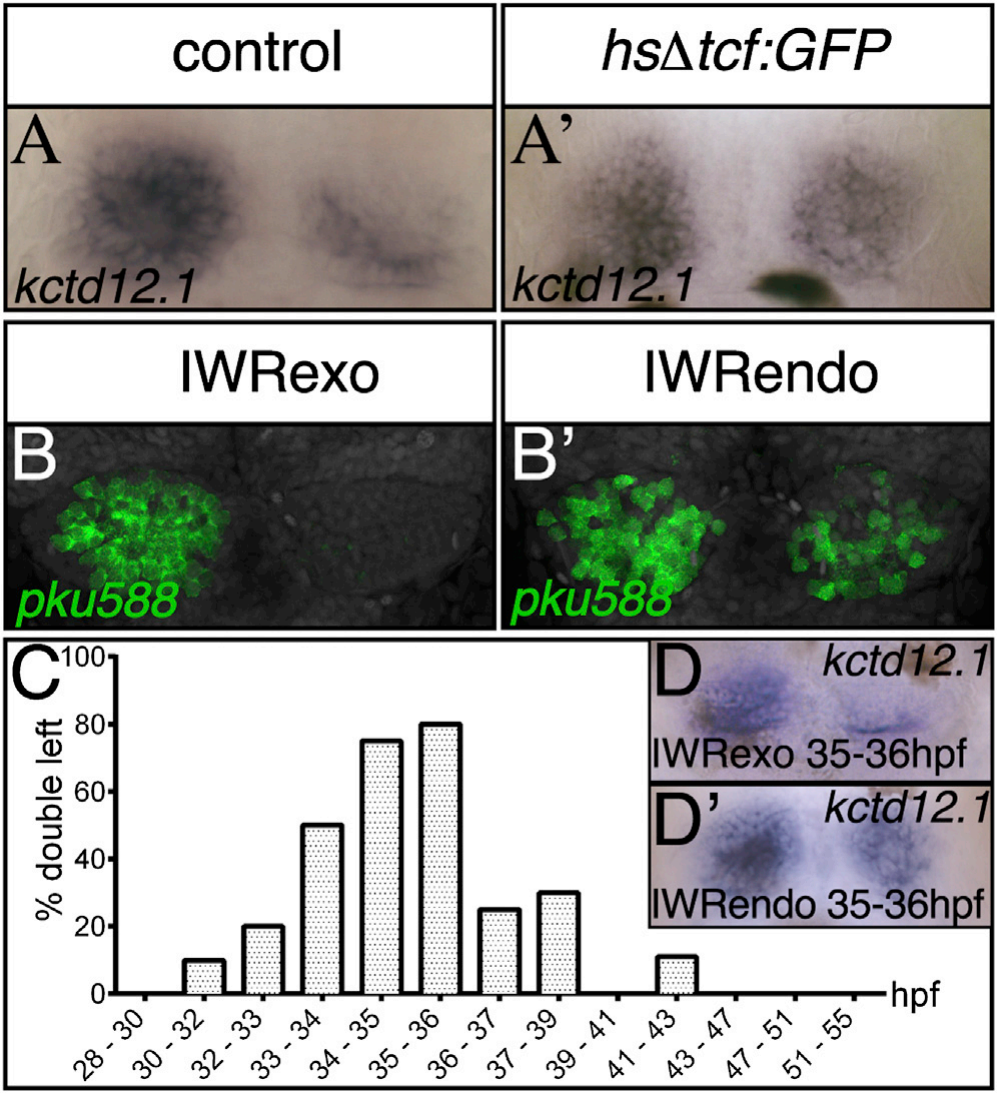
Wnt Signaling Is Required to Suppress dHbl Character in Right-Sided Neurons at the Onset of Tcf7l2 Expression (A–B′, D, and D′) Dorsal views with anterior to the top of 4 dpf embryos showing the upregulation of *kctd12.1* expression in the right habenula of a heat-shock-treated GFP^+^ Tg(hsp70:Δtcf-GFP) embryo (A′). The *pku588* transgene and *kctd12.1* are upregulated on the right side in IWRendo-treated embryos (B′ and D′). Control embryos were heat shocked or treated with IWRexo (A, B, and D). (C) Graph showing timing of IWRendo treatment and percentage of embryos with increased *kctd12.1* expression in the right habenula. See also Figure S5 and Table S4.

The results above suggest that Tcf7l2 function promotes dHbm fate (normally predominant on the right) and that dHb neurons adopt dHbl fate (normally predominant on the left) in absence of Tcf7l2 activity. In support of this idea, heat shock activation of a truncated Tcf that is believed to function as a dominant-negative form [32] at stages just prior to Tcf7l2 expression in dHb neurons results in an increased number of cells expressing the dHbl marker *kctd12.1* on the right side (Figures 7A, 7A′, and S5B and Table S4). Similarly, stabilization of Axin1 (and presumably suppressing Wnt signaling) with the drug IWRendo [20, 33] results in more cells expressing *kctd12.1* and *pku588* on the right, particularly when applied around the time of initial expression of neuronal Tcf7l2 and birth of dHb neurons (Figures 7B–7D′ and S5D and Table S4). Although both approaches to transiently interfere with Wnt signaling increased dHbl markers on the right, unlike in *tcf7l2* mutants, they had little or no effect on the predominantly right-sided expression of *kctd8* and the *hsp70-brn3a:GFP* transgene (Figure S5 and Table S4; n_total_ = 46/48).

These results suggest that reducing Wnt signaling around the time that dHb neurons are born leads to right-sided dHb neurons expressing dHbl markers. This is consistent with the data above suggesting that Tcf7l2 functions in newborn neurons to impart subnuclear character.

## Discussion

In this study, we identify Tcf7l2 as a critical regulator of brain asymmetry that mediates the elaboration of distinct differentiated character for equivalent neurons on the left and right sides of the brain. Tcf7l2 is expressed in nascent dHb neurons, which may all initially have the potential to develop with either dHbm or dHbl character. On the right side, Tcf7l2 cell/lineage autonomously promotes the elaboration of dHbm character typical of most right-sided neurons. On the left, the parapineal suppresses this Tcf7l2 dependent process, thereby enabling the acquisition of dHbl character typical of most left-sided neurons. Our study provides major insights into the signaling pathways and mechanisms by which equivalent neurons on left and right sides of the vertebrate central nervous system can acquire distinct differentiated character.

### Lateralized Activation of Tcf7l2 Signaling Promotes Asymmetric Neuronal Differentiation

Tcf7l2 is expressed in most, maybe all, dHb neurons on both left and right sides of the brain around their time of birth. In absence of Tcf7l2 function, we see no overt changes in the timing or patterns of neurogenesis in the dHb or in the numbers of neurons generated. Consequently, Tcf7l2 has no overt role in dHb neurogenesis per se and instead regulates the differentiated character of the dHb neurons that are generated.

Although it may initially seem counterintuitive that a transcription factor expressed in neurons on both left and right is a key regulator of asymmetric fate selection by the cells, bilateral expression is actually a prerequisite for such a function. Through being expressed in all neurons, Tcf7l2 can act as a regulatory switch for asymmetric fate selection. Essentially, it can function as the substrate upon which lateralized signals act to impart differentiated character to the dHb neurons on left and right sides of the brain. Conversely, any transcriptional regulator expressed only in dHbl or dHbm neurons could only function as an effector of that specific differentiated identity, downstream of whatever factors mediate fate selection.

How then could the presence of Tcf7l2 have different consequences on the left and right? In the Wnt pathway, Tcf family proteins are context-dependent transcriptional regulators [24]. In absence of pathway activation, they recruit proteins that establish repression at target sites, whereas when the pathway is active, they associate with β-catenin to activate target gene expression.

As in *tcf7l2* mutants, transient suppression of Wnt signaling during the period of dHb neuron generation leads to upregulation of dHbl markers in right-sided neurons, whereas in *axin1* mutants in which Wnt signaling is enhanced, dHbl markers are reduced and dHbm markers expanded in a Tcf7l2-dependent manner. Together, these observations suggest that in the wild-type situation, Wnt/β-catenin signaling acts through Tcf7l2 to impart dHbm identity, mostly to neurons on the right. Consequently, we propose that the functional consequences of the presence of Tcf7l2 in dHb neurons may depend on the status of Wnt pathway activation upstream of this transcriptional effector. Wnt pathway activation is sensed by Tcf7l2 and is translated into a selection between either dHbl or dHbm subnuclear identity by the differentiating neurons. This mode of function for Tcf7l2 differs from that proposed for the ventral habenula [34], within which it appears to have a more canonical Wnt pathway role in promoting neurogenesis rather than in regulating differentiated character of the neurons generated.

### Parapineal Signals Oppose Tcf7l2-Dependent Acquisition of dHbm Fate on the Left Side of the Brain

Our results suggest that LR asymmetries in relative frequency of dHbm and dHbl neurons are caused by a LR asymmetry in Tcf7l2 signaling in nascent habenular neurons. What then accounts for the differential activation of Tcf7l2 signaling on the left and right? Our data support the idea that this asymmetry is brought about by the parapineal preventing pathway activation in nascent neurons on the left. The parapineal is required for elaboration of dHbl character, and if it is ablated, most left-sided neurons adopt a dHbm fate such that left and right habenulae become largely symmetric with right-sided character [9, 16, 18, 21]. This is the opposite phenotype to loss of Tcf7l2 function, suggesting that parapineal signals oppose the function of Tcf7l2 in nascent neurons. Indeed, in absence of Tcf7l2 function, neurons on both left and right adopt dHbl identity independent of the presence of the parapineal. Consequently, the role of the parapineal is rendered redundant in absence of Tcf7l2, and its normal role must therefore be to counteract Tcf7l2-dependent signaling in nascent neurons on the left. How it does this is unknown. Certainly, the parapineal appears not to have any effect on *tcf7l2* gene or protein expression (data not shown). One possibility is that the parapineal somehow suppresses the level of Wnt pathway activation experienced by nascent habenular neurons. This possibility is supported by the observation that activation of Wnt signaling in *axin1* mutants phenocopies parapineal ablation with respect to neurons bilaterally adopting dHbm identity.

In wild-type embryos, the left habenula contains a small proportion of right-typical dHbm neurons that innervate the ventral IPN [18]. In contrast, in *tcf7l2* mutants, habenular axons from both sides appear to exclusively innervate the dorsal IPN, suggesting there might be no (or undetectably few) fully differentiated dHbm neurons with ventral-IPN-directed axons on either side. Also, some dHbm marker genes are not expressed at all in mutants, and there is no subnuclear division of habenular neuropil domains. Consequently, in the *tcf7l2* mutant, there appears to be a complete loss of the ability to activate the genetic program for dHbm neuron differentiation, thus leading to an exaggerated symmetric left-typical phenotype. In contrast, in the wild-type, we suggest that the parapineal does not fully antagonize the dHbm promoting activity of Tcf7l2, with the consequence that there is differentiation of some dHbm neurons on the left. In this regard, it has recently been observed that when the parapineal is enlarged, more dHb neurons differentiate with dHbl character [35], supporting the idea that parapineal signals are regionally restricted in wild-type conditions.

### Tcf7l2 Functions Independently of Notch-Dependent Neurogenesis

Production of dHb neurons starts and peaks a little earlier on the left than on the right, and dHbl neurons tend to be produced prior to dHbm neurons [22]. Furthermore, the loss of Notch signaling in *mindbomb* (*mib*) mutants induces premature habenular neurogenesis and results in most neurons elaborating dHbl identity on both the left and right [22]. These observations have led to the proposal that asymmetries in Notch-signaling-dependent neurogenesis underlie the development of asymmetric pools of dHbm and dHbl neurons on the left and right. In such a scenario, reduced Notch signaling on the left at early stages results in more dHbl neurons being born; conversely, higher levels of Notch signaling on the right at early stages would keep precursors dividing until later stages, when they would tend to generate dHbm neurons. If and how this asymmetry relies on parapineal signaling is not known. Is this model consistent with our observations on the roles for Tcf7l2 in assigning dHbl versus dHbm identity?

In absence of Tcf7l2 function, neither the timing nor the initial asymmetry in habenular neurogenesis is affected. This suggests that modulations to Tcf7l2 signaling do not significantly impact on Notch-signaling-dependent neurogenesis. This also implies that the symmetric left-typical phenotype of *tcf7l2* mutants is unlikely to be explained by a major (*mib*-like) Notch-pathway-dependent acceleration in the production of neurons.

Rather than Notch signaling directly impacting the differentiated character of dHb neurons, it is perhaps more likely that the environment into which the neurons are born changes over time. If so, it would be these changes in the environment that shift the probability of whether dHb neurons born at different times adopt dHbl or dHbm identities. Given our results, one feature of the environment that could change over time is the level of Wnt signaling seen by neurons as they exit the cell cycle.

Overall, we suggest that Tcf7l2- and Notch-dependent neurogenesis act in parallel, with levels of activation of both pathways contributing to the asymmetric subnuclear organization of the dHb. The critical feature in assignment of dHbl and dHbm character is the level of Tcf7l2-dependent signaling to which the neuron is exposed around the time of its birth. The Notch pathway could indirectly influence this by altering the timing of neurogenesis (and consequently the environment into which the neurons are born), whereas Tcf7l2 directly affects this by enabling neurons to correctly perceive environmental signals. This model suggests an alternative or additional role for the parapineal. If the parapineal influences the Notch-dependent timing of neurogenesis, it could affect the environment into which the neurons are born, biasing their selection of dHbl or dHbm fate. Parapineal ablation does not appear to have an overt effect on the asymmetric production of the first dHb neurons [23], but it is not known whether later asymmetries in neurogenesis are affected.

### Differentiated Character of Habenular Projection Neurons Is Specified around the Time of Their Birth

Our data suggest that most, maybe all, dHb neurons initially have the potential to elaborate either dHbl or dHbm character and that this fate selection occurs very late in progression from neural precursor to differentiated neuron. Although many fate decisions are taken much earlier in neural lineages, there are other examples of factors acting upon postmitotic neurons to influence their differentiated character. For instance, in the spinal cord, target-derived cues provided by afferent input onto postmitotic motor neurons regulate late aspects of motor neuron differentiation [36]. It is believed that this type of communication between different neuronal components within the circuit helps to ensure correct circuit differentiation and function. Although details may be different in the habenulae, the concept is similar. Parapineal cells communicate to habenular neurons, their potential synaptic partners, thereby influencing their program of differentiation [5] and the targeting of their axon terminals within the interpeduncular nucleus.

The Wnt pathway is remarkable in the variety of roles it is proposed to play in neural development, and indeed, we have defined entirely different roles for Wnt signaling in early steps of nervous system asymmetry [13, 15]. The pathway has previously been implicated in determination of neuronal identity, perhaps most precisely in studies of neurogenesis in worms [37, 38]. In *Caenorhabditis elegans*, the Wnt asymmetry pathway acts at various steps within lineages, regulating the expression of homeodomain transcription factors that eventually control neuronal differentiation. Ceh-10, for instance, is activated by Wnt signaling in one of the two daughter cells after the terminal division of a neural progenitor and regulates terminal differentiation genes in postmitotic neurons [39]. Tcf does not associate with β-catenin in the other daughter cell, Ceh-10 is inhibited, and the cell adopts a different identity. Based on these and other data, a binary decision model has been proposed to explain the transition between regulatory states of cells [38]. According to this model, the status of Wnt activity within the two daughter cells leads to selection of alternate terminal differentiation programs, resulting in cell fate diversity. This mode of generating cell diversity has also been demonstrated in the annelid *Platynereis* [40], but the situation in vertebrates is less clear as Wnt/β-catenin levels seem to be most important for neural precursors to chose between proliferation and differentiation states [38, 41].

We show here that the activity of Tcf7l2 in differentiating habenular neurons is an important determinant of their fate. This suggests that a Tcf-dependent binary decision model may also apply to fate decisions made during vertebrate neuronal development.

In summary, our study suggests an intuitively simple way in which equivalent neurons on left and right sides of the brain can differentiate with different properties by assessing their environment at the time of their birth. Varying the pertinent environmental signals may impact upon the extent of the asymmetry potentially contributing to variability between species and between individuals of the same species [1].

## Experimental Procedures

### Fish Lines and Maintenance

Zebrafish were maintained according to standard procedures. For inhibition of pigmentation, embryos were incubated in 0.2 mM 1-phenyl-2 thiourea (PTU). Lines used and genotyping assays are indicated in Supplemental Experimental Procedures. All animal procedures were approved by local review boards.

### Whole-Mount In Situ Hybridization and Antibody Labeling

Antisense probes were generated using digoxigenin and fluorescein RNA labeling kits (Roche), and 5-bromo-4-chloro-3′-indolyphosphate and nitro-blue tetrazolium chloride, BM Purple AP Substrate (Roche), or Fast Red (Sigma) were used in standard protocols [42].

The antibodies used were anti-GFP antibody (1:1,000, Torrey Pines Biolabs), anti-RFP antibody (1:1,000, MBL PM055), anti-Human TCF3,4 antibody (1:400, Biomol, clone 0.T.148), anti-HuC/D antibody (1:500, Molecular Probes), Alexa Fluor 488-conjugated and Alexa Fluor 647-conjugated secondary antibodies (1:200, Molecular Probes), and anti-SV2 antibody (1:250, DSHB). Stainings were performed according to standard procedures [43]. For nuclear stainings, embryos were incubated in PBS, 0.8% Triton X-100, and 1% BSA containing Sytox orange (1:10,000, Invitrogen), Topro3 (1:1,000, Molecular Probes), or DAPI (10 μg/ml, Thermo Scientific).

It is likely that the anti-Human TCF3,4 antibody predominantly recognizes Tcf7l2 as immunoreactivity is lost in *tcf7l2*^u754^ mutants, but not in *hdl/tcf7l1a*^m881^ maternal zygotic mutants. Immunoreactivity against a protein smaller in size than full-length Tcf7l2 is maintained in *tcf7l2*^u763^ and *tcf7l2*^exl^ alleles. We think that this is most likely due to transcription from an alternative start site previously described in mice [44], located in the zebrafish genome between exons 4 and 5, that is disrupted by the *u754* mutation. All three mutant alleles show a symmetric habenular phenotype, suggesting that any truncated protein made in the *u763* and *exl* alleles has no significant role in habenular asymmetry.

### Labeling of Habenular Efferent Projections

Embryos were fixed overnight at 4°C in 4% paraformaldehyde plus 4% sucrose (m/v) in PBS. After brain dissection, fluorescent carbocyanine dyes (1,1-dioctadecyl-3,3,3,3-tetramethylindocarbocyanine perchlorate [DiI] and 1,1′-dioctadecyl-3,3,3′,3′-tetramethylindodicarbocyanine, 4-chlorobenzenesulfonate salt [DiD; Sigma]) were applied with tungsten needles as described previously [17]. Brains were incubated overnight at 4°C and imaged.

### IWR, Heat Shock, and BrdU Treatments

Dechorionated embryos were incubated in IWR endo or IWR exo (diastereometric form of IWR endo)-containing solutions (0.1 mM in E3 embryo medium/1% DMSO, Sigma) starting at 32 hpf for 12 hr or as indicated.

Heat shocks of Tg(hsp70:Δtcf-GFP)^w26^ embryos were performed at 33 hpf and 34 hpf for 30 min at 40°C. BrdU incorporation was performed as previously described [45].

### Transplantation Experiments

Embryos from Et(gata2a:EGFP)^pku588^ × *tcf7I2*^+/exl^ incrosses were injected with mRNA encoding nuclear RFP (40–50 pg per embryo) at the one-cell stage. Twenty to thirty RFP^+^ cells were transplanted from the apical region of midblastula donor embryos (high to sphere stage) into the apical, brain-forming region of wild-type hosts. Donor embryos were subsequently genotyped. Host embryos were fixed at 54 or 58 hpf, and RFP and enhanced GFP were detected with antibodies as above.

### Laser Cell Ablation

Ablations were performed using two-photon excitation at 800 nm with a Chameleon tunable laser (Coherent)-A1R MP (Nikon) setup, with a 40× water-immersion long-working distance 40×WI λS numerical aperture 1.15 Nano Crystal Coated objective. For acquisition and ablation, 29%–31% laser power was used, which corresponds to 9.5 mW of infrared laser power behind the objective lens. Embryos were subsequently imaged and fixed for labelings.

### Microscopy and Image Manipulation

For transmitted light pictures, larvae were mounted in glycerol and imaged using differential interference contrast optics (Leica CTR6000; 20× and 40× objectives). For confocal microscopy, heads were mounted in 1.2% low-melt agarose in glass-bottom dishes (MatTek or LabTek). Fluorescence was imaged by confocal laser scanning microscopy (Leica TCS SP5 and Leica TCS SP8) using a 40× oil-immersion objective (40× 1.3 Oil DIC III) or a 25× water-immersion objective (25 × 0.95), and z stacks were acquired in 0.75–2 μm intervals. 3D reconstructions and maximum-intensity projections were generated from stacks of images with Volocity (Improvision) and ImageJ (NIH). Pseudocoloring of flattened images was performed with Photoshop CS4 (Adobe).

### Cell Counting and Statistical Analysis

Cell counting was performed from confocal data sets with optical planes acquired at 3 μm intervals. 3D reconstructions of imaged volumes were examined to identify individual cells. For anti-Tcf7l2- and anti-HuC/D-labeled habenular cells, the outer cell layer was counted, and the average, SD, and percentages were calculated using Prism 4 (GraphPad Software). For BrdU-labeled and transplanted *Et(gata2a:EGFP)*^*pku588*^ cells, the entire dHb was counted.

We performed ANOVA analyses to assess the effects of age, genotype, and laterality (left versus right) on numbers of HuC/D, *HuC:GFP*, and BrdU/*pku588* cells (Figure 3; MATLAB, Mathworks). Tukey-Kramer post tests, corrected for multiple comparisons, were used to evaluate statistical significance of specific pairwise comparisons, as indicated in the Results.

The effects of genotype and laterality on the numbers of transplanted cells expressing *pku588* were estimated by fitting of a generalized linear model to our data, with binomial outcome and a logit link function (MATLAB function “GeneralizedLinearModel.stepwise”).

## Author Contributions

M.C. and S.W.W. conceived and planned the study and wrote the paper with help from I.H.B., G.G., H.L.S., and A.F. U.H. helped to design and carried out phenotype analysis of mutants and parapineal ablations. H.L.S. identified and contributed to phenotype analysis of two novel *tcfl2* mutant alleles, and G.G. performed BrdU and transplantation experiments. I.H.B. and A.F. performed neuroanatomical analyses, and I.H.B. carried out statistical analyses. All other authors contributed to experimental design, screening, phenotype analysis of mutants, and/or generation of reagents.

## Supplemental Information

Document S1. Supplemental Experimental Procedures, Figures S1–S5, and Tables S1–S4

Movie S1. 3D Reconstruction of the Habenular Neuropil, Related to Figure 2 Dorsal view with anterior to the top of a 4 dpf wild-type embryo stained for acetylated tubulin (cyan) and SV2 (red). The 3D reconstruction visualizes the subnuclear neuropil domains.

Movie S2. The Habenular Neuropil of *tcf7l2* Mutants Is Symmetric and Not Devided into Subnuclear Domains, Related to Figure 2 3D reconstruction of a dorsal view with anterior to the top of a 4 dpf *tcf7l2* mutant embryo stained for acetylated tubulin (cyan) and SV2 (red).

Document S2. Article plus Supplemental Information
