# Supplementary material for: Tcf7l2 Is Required for Left-Right Asymmetric Differentiation of Habenular Neurons
Source: Curr Biol. 2014 Oct 6;24(19):2217–27. doi: 10.1016/j.cub.2014.08.006 (PMC4194317; doi:10.1016/j.cub.2014.08.006)
Supplement: Document S1. Supplemental Experimental Procedures, Figures S1–S5, and Tables S1–S4 [file mmc1.pdf]

Current Biology, Volume *24*

Supplemental Information

Tcf7l2 Is Required for Left-Right Asymmetric Differentiation of Habenular Neurons

Ulrike Hüsken, Heather L. Stickney, Gaia Gestri, Isaac H. Bianco, Ana Faro, Rodrigo M. Young, Myriam Roussigne, Thomas A. Hawkins, Carlo A. Beretta, Irena Brinkmann, Alessio Paolini, Raquel Jacinto, Shahad Albadri, Elena Dreosti, Matina Tsalavouta, Quenten Schwarz, Florencia Cavodeassi, Anukampa K. Barth, Lu Wen, Bo Zhang, Patrick Blader, Emre Yaksi, Lucia Poggi, Mihaela Zigman, Shuo Lin, Stephen W. Wilson, and Matthias Carl

Supplemental Figures

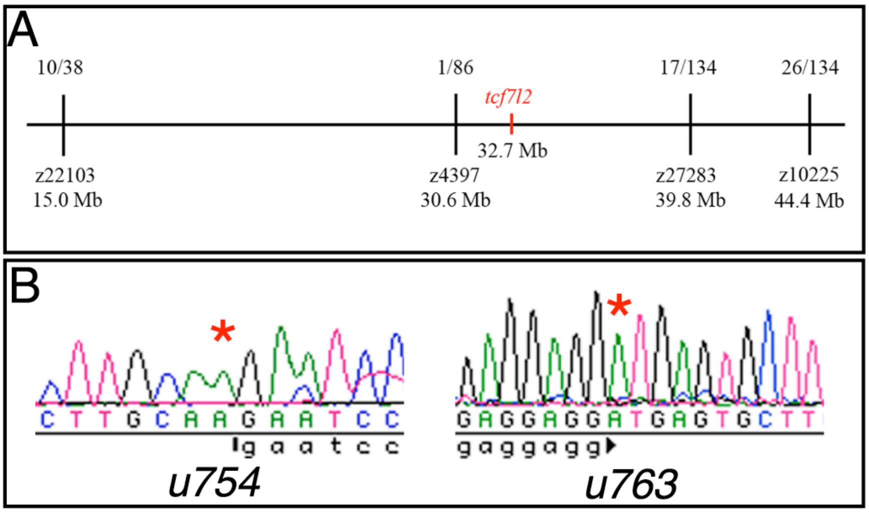

Figure S1

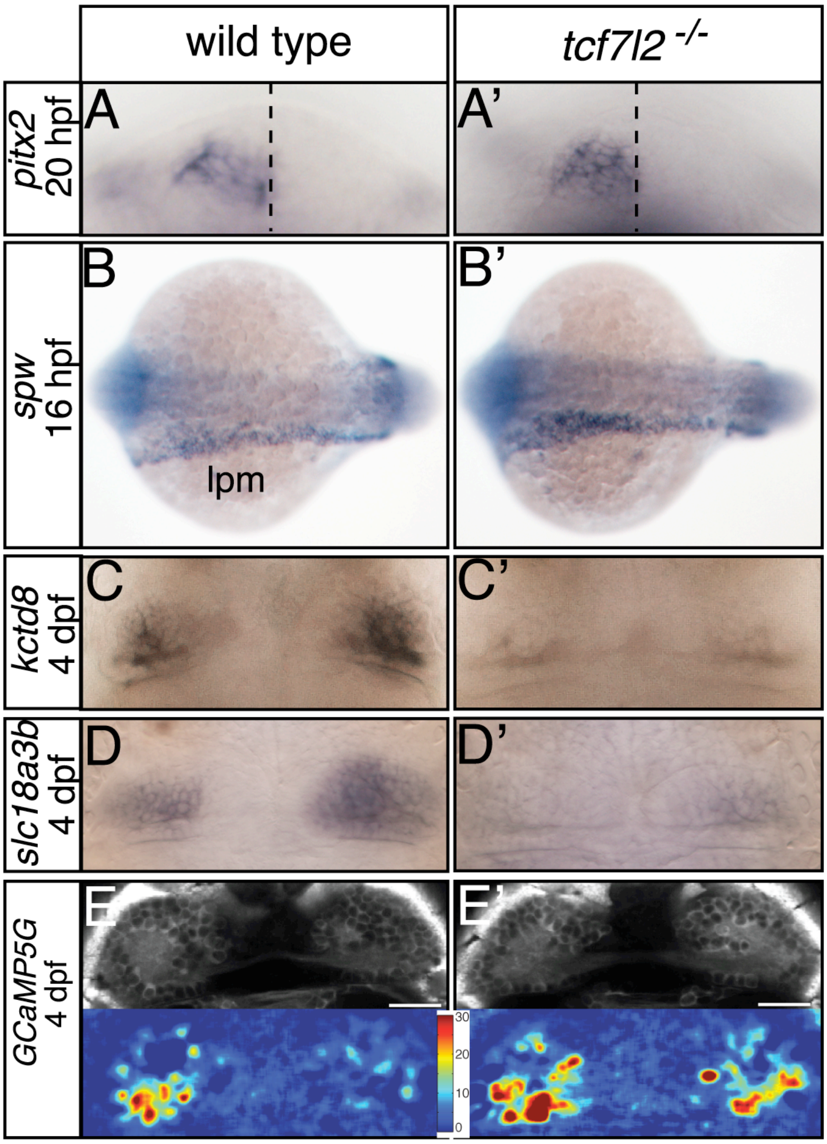
wild type
tcf7l2 -/-
pitx2
20 hpf
A
A'
spw
16 hpf
B
B'
lpm
kctd8
4 dpf
C
C'
slc18a3b
4 dpf
D
D'
GCaMP5G
4 dpf
E
E'
30
20
10
0

Figure S2

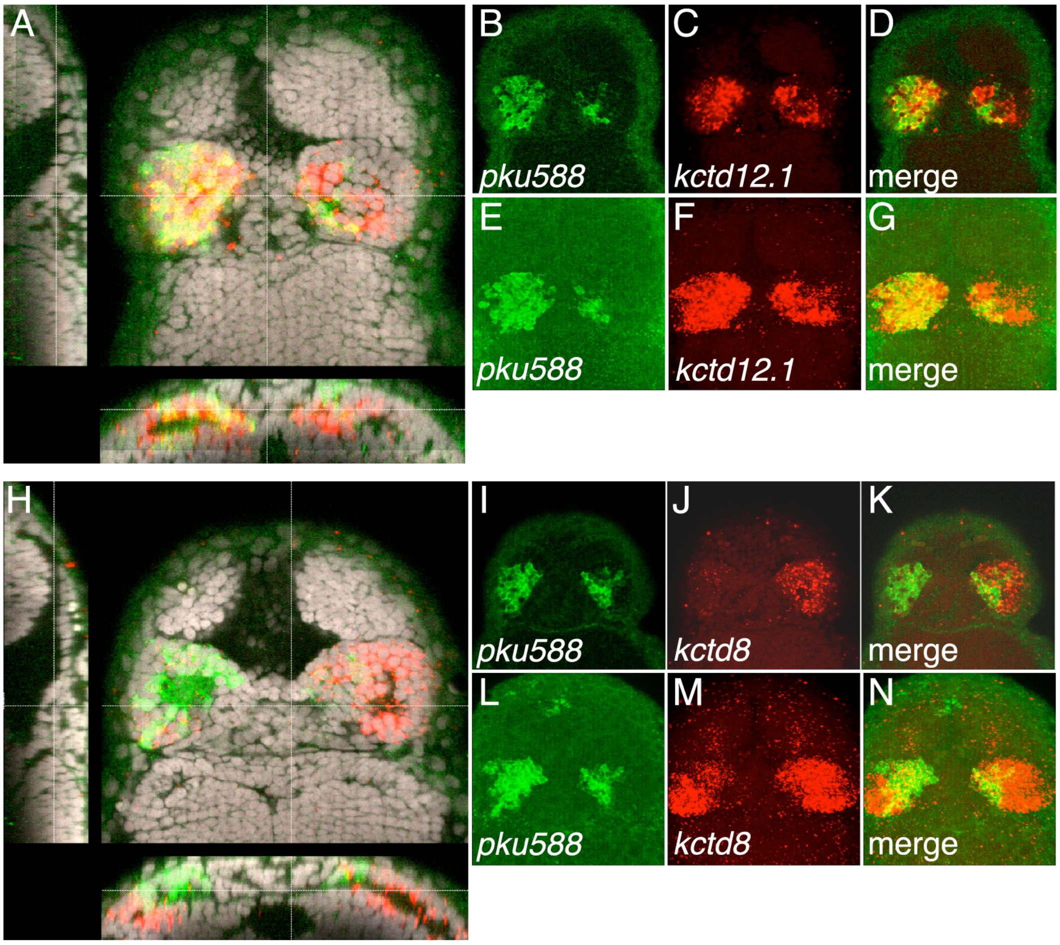
A
B
C
D
pku588
kctd12.1
merge
E
F
G
pku588
kctd12.1
merge
H
I
J
K
pku588
kctd8
merge
L
M
N
pku588
kctd8
merge

Figure S3

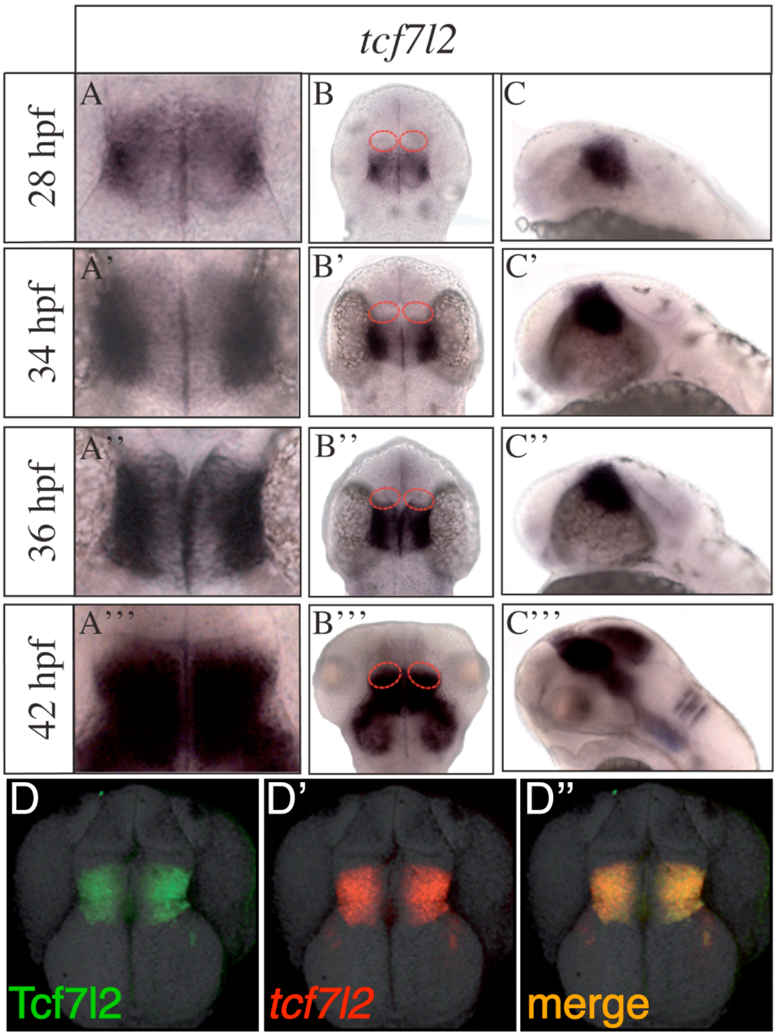
tcf7l2
28 hpf
34 hpf
36 hpf
42 hpf
A
B
C
A’
B’
C’
A’’
B’’
C’’
A’’’
B’’’
C’’’
D
D’
D’’
Tcf7l2
tcf7l2
merge

Figure S4

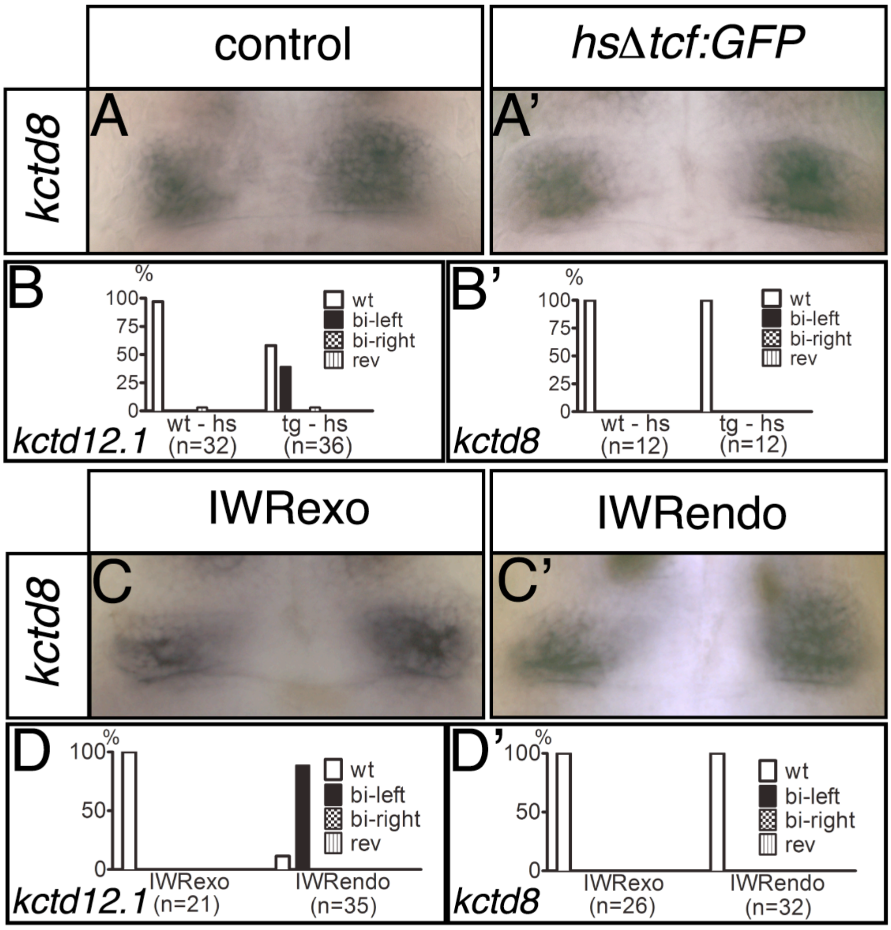
control
hsΔtcf:GFP
kctd8
A
A'
B
%
100
75
50
25
0
wt
bi-left
bi-right
rev
kctd12.1
wt - hs
(n=32)
tg - hs
(n=36)
B'
kctd8
wt - hs
(n=12)
tg - hs
(n=12)
IWRexo
IWRendo
C
C'
D
100
50
kctd12.1
IWRexo
(n=21)
IWRendo
(n=35)
D'
kctd8
IWRexo
(n=26)
IWRendo
(n=32)

Figure S5

Supplemental Figure Legends

Figure S1, related to Figure 1. Identification of two novel *tcf7l2* mutant alleles.

(A) The *u754* mutation maps to a 9.2 Mb interval on LG12 between markers z4397 and z27283 that contains the *tcf7l2* gene.

(B) *u754* mutants have an A to G substitution in the splice acceptor of exon 6 and *u763* mutants have a G to T substitution at the splice donor of exon 2.

Figure S2, related to Figure 2. *tcf7l2* mutants develop symmetric habenulae with left-sided character.

(A,A') Frontal views of 20 hpf embryos with the dotted line marking the midline of the dorsal diencephalon. Asymmetric expression of *pitx2* in the brain is unaffected in the *tcf7l2* mutant.

(B,B') Dorsal views of 16 hpf embryos focussed on the trunk region with anterior to the left. *spw* expression in the LPM is unaffected in the *tcf7l2* mutant.

(C-E') Dorsal views of the habenulae in 4 dpf embryos with anterior to the top showing markers indicated to the left. (E,E') Example of a two-photon image of a single z-plane of the dHb (14 µm below the skin) in Tg(elav3:GCaMP5G) transgenic embryos. Colour-coded calcium signals in response to a non-lateralized light stimulus (bottom). Each panel is an average of two light stimulus trials. The relative change in fluorescence ($\Delta F/F$) is expressed as a percentage. The habenulae of all *tcf7l2* mutants (n=8/8) showed bilaterally symmetric responses to light, while the responses in wildtypes are lateralized to the left (n=5/5). The scalebar corresponds to 20 µm.

lpm, lateral plate mesoderm.

Figure S3, related to Figure 2. The *gata2a:EGFP*^{pku588} (*pku588*) transgene is expressed in dHbl cells.

(A-N) Dorsal views of 3 dpf *pku588* transgenic embryos with anterior to the top. Expression of the dHbl marker *kctd12.1* overlaps with the *pku588* transgene (A-G) whereas transgene expression is mostly complementary to *kctd8* (H-N). (A,H) Sections of a D-V reconstruction; (B-D,I-K) sections and (E-G,L-N) maximum projections.

Figure S4, related to Figure 4. *tcf7l2* mRNA and Tcf7l2 protein is widely expressed in the diencephalon.

(A-C''') Dorsal and lateral views of *tcf7l2* mRNA expression in the diencephalon with anterior to the (A-B''') top or (C-C''') left at stages shown on left. Dotted circles mark the approximate habenula area.
(D-D'') Dorsal view with anterior to the top of a 36 hpf embryo showing overlapping expression of Tcf7l2 and *tcf7l2*. Cell nuclei are labelled with Sytox orange. Antibody specificity was further confirmed with western-blots using wildtype, *tcf7l2* and *tcf3* mutants (see experimental procedures and data not shown).

Figure S5, related to Figure 7. Inhibition of Wnt/β-catenin signalling increases the number of dHbl neurons in the right habenula.
(A,A',C,C') Dorsal views of the habenulae with anterior to the top of 4 dpf embryos.
(A,A') Embryos were heat shock treated at 33 and 34 hpf for 30 min at 40°C. Embryos were sorted for GFP expression; GFP negative embryos served as controls.
(C,C') Embryos were treated with IWRexo (control) or IWRendo for 16 hrs beginning at 32 hpf.
(B,B',D,D') Graphs illustrate the percentage of embryos showing wildtype (wt), reversed (rev), bilateral-left (bi-left) or bilateral-right (bi-right) marker gene expression as indicated.

Supplemental Tables

Table S1, related to Figure 2. ***tcf7l2*** **mutant analysis**

marker	wt	rev	bi-left	bi-right	absent	n
kctd12.1	0 (95)	0 (5)	100 (0)	0 (0)	0 (0)	42 (58)
cadps2	0 (86)	0 (0)	100 (14)	0 (0)	0 (0)	4 (7)
pku588	4 (98)	0 (2)	96 (0)	0 (0)	0 (0)	28 (47)
kctd12.2	0 (95)	0 (5)	0 (9)	100*(0)	0 (0)	17 (19)
kctd8	0 (91)	0 (9)	0 (0)	0 (0)	100*(0)	34 (22)
tag1	0 (100)	0 (0)	0 (0)	0 (0)	100 (0)	7 (12)
slc18a3b	0 (92)	0 (6)	0 (0)	0 (2)	100*(0)	15 (52)
brn3a:GFP	40 (94)	0 (0)	0 (0)	60* (6)	0 (0)	15 (17)
DiI/DiO	0 (100)	0 (0)	100***(0)	0 (0)	0 (0)	6 (7)
cxcr4b	98 (100)	0 (0)	0 (0)	0 (0)	2*(0)	43 (38)
gfi	100 (86)	0 (14)	0 (0)	0 (0)	0 (0)	3 (14)
otx5	100 (100)	0 (0)	0 (0)	0 (0)	0 (0)	5 (22)
foxD3:GFP	100 (100)	0 (0)	0 (0)	0 (0)	0 (0)	11 (14)
pitx2 – brain**	96	0	2	0	2	46
pitx2 – LPM**	96	0	0	0	4	46
fkd2 – liver**	94	0	6	0	0	31

All embryos were genotyped before labelling unless indicated otherwise; scores in columns 2-6 show percentages; numbers represent *tcf7l2* homozygous mutant embryos and numbers of sibling embryos are shown in parenthesis; * reduced expression; ** whole clutch; *** bilateral (bi) dorsal; rev, reversed.

Table S2, related to Figure 3 and 4. Tcf7l2 is expressed in dHb neurons and does not influence the timing of neurogenesis

marker/age	genotype	left	right	n
HuC/D@34hpf	wildtype	10.5±2.65	5±2.45	4
HuC/D@34hpf	*tcf7l2-/-*	11.5±1.29	5.75±2.22	4
HuC/D@36hpf	wildtype	14.8±3.77	9.6±3.05	5
HuC/D@36hpf	*tcf7l2-/-*	14.6±3.36	8.6±1.52	5
HuC:GFP@39hpf	wildtype	17.67±4.82	13.22±4.47	9
HuC:GFP@39hpf	*tcf7l2-/-*	17.67±4.69	13.89±4.48	9
HuC:GFP@48hpf	wildtype	33.5±7.18	26.5±6.33	10
HuC:GFP@48hpf	*tcf7l2-/-*	34.1±8.01	29±9.84	10
cxcr4b@32hpf	wildtype	10.33±4.59	4.83±3.66	6
cxcr4b@32hpf	*tcf7l2-/-*	11.4±4.88	6±4.64	5
BrdU@32hpf, counted @3dpf	wildtype	43.8±12.2	29.7±12.3	6
BrdU@32hpf, counted @3dpf	*tcf7l2-/-*	47.3±8.14	37.7±6.11	3
BrdU@32hpf, counted @5dpf	wildtype	39.4±9.62	23.5±4.79	10
BrdU@32hpf, counted @5dpf	*tcf7l2-/-*	40.8±5.54	26.6±4.04	5
Tcf7l2@34hpf	wildtype	0±0	0±0	10
Tcf7l2@35hpf	wildtype	4.8±1.32	1.5±0.97	10
Tcf7l2@36hpf	wildtype	11.9±2.18	7.5±1.9	10
Tcf7l2@37hpf	wildtype	16.6±3.95	13.7±4.37	10
Tcf7l2@38hpf	wildtype	23.1±5.65	20.5±4.35	10
Tcf7l2@40hpf	wildtype	34±5.91	33.8±8.18	10
Tcf7l2+HuC:GFP @40hpf	wildtype	27.3±9.99	20.8±8.52	10

Values given as mean ± standard deviation. All *tcf7l2*$^{+/-}$ incross derived embryos were genotyped.

Table S3, related to Figure 6. *tcf7l2 /axin1* double mutant analysis

marker	genotype	wt	rev	bi-left	bi-right	absent	n
kctd12.1	wt	31	6	0	0	0	33
	axin1$^{-/-}$	0	0	0	100	0	32
	tcf7l2$^{-/-}$	0	0	100	0	0	26
	tcf7l2$^{-/-}$; axin1$^{-/-}$	6	0	94	0	0	16
kctd8	wt	91	9	0	0	0	22
	axin1$^{-/-}$	5	0	0	95	0	21
	tcf7l2$^{-/-}$	0	0	100*	0	0	21
	tcf7l2$^{-/-}$; axin1$^{-/-}$	0	0	100*	0	0	10

All embryos were genotyped and scores in columns 3-7 show percentages; * reduced expression; bi, bilateral; rev, reversed.

Table S4, related to Figure 7. Transient interference with Wnt/β-catenin signalling

treatment and hpf	wt	rev	bi-left	bi-right	absent	n
IWRendo 32-48 kctd12.1	11	0	89	0	0	35
IWRexo 32-48 kctd12.1	100	0	0	0	0	21
IWRendo 32-48 kctd8	100	0	0	0	0	32
IWRexo 32-48 kctd8	100	0	0	0	0	26
IWRendo 32-48 pku588	15	0	85	0	0	27
IWRexo 32-48 pku588	100	0	0	0	0	10
IWRendo 32-48 brn3a:GFP	88	0	0	12	0	16
IWRexo 32-48 brn3a:GFP	100	0	0	0	0	14
IWRendo 28-30 kctd12.1	100	0	0	0	0	20
IWRendo 30-32 kctd12.1	90	0	10	0	0	20
IWRendo 32-33 kctd12.1	80	0	20	0	0	20
IWRendo 32-33 kctd12.1	80	0	20	0	0	20
IWRendo 32-34 kctd12.1	20	0	80	0	0	10
IWRendo 34-35 kctd12.1	25	0	75	0	0	20
IWRendo 34-36 kctd12.1	10	0	90	0	0	10
IWRendo 35-36 kctd12.1	20	0	80	0	0	20
IWRendo 36-37 kctd12.1	75	0	25	0	0	20
IWRendo 36-38 kctd12.1	30	0	70	0	0	10
IWRendo 37-39 kctd12.1	70	0	30	0	0	20
IWRendo 39-41 kctd12.1	100	0	0	0	0	20
IWRendo 41-43 kctd12.1	91	0	9	0	0	11
IWRendo 43-47 kctd12.1	100	0	0	0	0	10
IWRendo 47-51 kctd12.1	100	0	0	0	0	12
IWRendo 51-55 kctd12.1	100	0	0	0	0	10
hsΔtcf 33+34 kctd12.1	58	3	39	0	0	36
hsΔtcf 33+34 kctd12.1 control	97	3	0	0	0	32
hsΔtcf 33+34 kctd8	100	0	0	0	0	12
hsΔtcf 33+34 kctd8 control	100	0	0	0	0	12

All embryos were analysed at 4 dpf and scores in columns 2-6 show percentages; hsΔtcf control embryos did not show flurescence after heat shock treatment; bi, bilateral; rev, reversed.

Supplemental Experimental Procedures

Fish lines

AB and *tupl* wildtype lines, the $tcf7l2^{exl}$ [S1], *u763* and *u754 tcf7l2* mutant alleles and *masterblind/axin1* (mbl^{tm213}) [S2] and $tcf7l2^{exl}$ x mbl^{tm213} mutant lines were used. $tcf7l2^{u763}$ and $tcf7l2^{u754}$ were generated in an ENU-mutagenesis screen [S3]. The following transgenic lines were used: Tg(HuC:GFP) [S4], Tg(elavl3:GCaMP5G)^{2} [S5], Et(gata2a:EGFP)^{pku588} derived from an enhancer trap screen [S6], Tg(hsp70-brn3a:GFP)^{rw0110b} [S7], Tg(hsp70:Δtcf-GFP)^{w26} [S8], Tg(foxD3:GFP)^{zf104}; Tg(flh:eGFP)^{U711} [S9, S10], $tcf7l2^{exl}$ x Et(gata2a:EGFP), $tcf7l2^{exl}$ x Tg(hsp70-brn3a:GFP), $tcf7l2^{exl}$ x Tg(elavl3:GCaMP5G)^{2}, mbl^{tm213} x Tg(foxD3:GFP); Tg(flh:eGFP), Et(gata2a:EGFP); Tg(foxD3:GFP); Tg(flh:eGFP), $tcf7l2^{exl}$ x Et(gata2a:EGFP); Tg(foxD3:GFP); Tg(flh:eGFP).

Genetic mapping and genotyping

The $tcf7l2^{u754}$ mutation was mapped by bulked segregant analysis [S11]. Genomic PCR and sequencing of exon VI and VII with primers F-5;- GTGAATGTGTGTGTCCATCCA and R- 5'- TGAGATGGCCTTCTGCTAC revealed a splice acceptor lesion in exon VI of $tcf7l2^{u754}$; mutants were subsequently genotyped with the DCAPS primers F-5'-GTGGAGGCCTTGGGGTTC-3' and R-5'-TGTTCAATTGTGTATCTCACAAAGG-3' and the restriction enzyme NlaIV. The lesion in the splice donor of exon II in $tcf7l2^{u763}$ mutants was identified by genomic PCR and sequencing with the primer pair F-5'-TTCAATGCATTCCCTGTGTT-3', R-5'-GTCCTGTGCAAAAACAAAAAG-3'; the same primer pair was then used with restriction enzyme HlhI for genotyping. $tcf7l2^{exl}$ mutant embryos were genotyped by genomic PCR using primers F-5′-GACGAAGGCGAGCAGGAGG-3′ and R-5′-GAAATGCAACGAAACACAGGGAATGC-3′ followed by digestion with BsajI restriction enzyme.

Functional imaging and sensory stimulation

Functional imaging and light stimulations were performed as described [S12].

Supplemental References

S1. Muncan, V., Faro, A., Haramis, A.-P.G., Hurlstone, A.F.L., Wienholds, E., van Es, J., Korving, J., Begthel, H., Zivkovic, D., and Clevers, H. (2007). T-cell factor 4 (Tcf7l2) maintains proliferative compartments in zebrafish intestine. EMBO reports *8*, 966-973.

S2. Heisenberg, C.P., Houart, C., Take-uchi, M., Rauch, G.J., Young, N., Coutinho, P., Masai, I., Caneparo, L., Concha, M.L., Geisler, R., et al. (2001). A mutation in the Gsk3-binding

domain of zebrafish Masterblind/Axin1 leads to a fate transformation of telencephalon and eyes to diencephalon. Genes & Development *15*, 1427-1434.

S3. Valdivia, L.E., Young, R.M., Hawkins, T.A., Stickney, H.L., Cavodeassi, F., Schwarz, Q., Pullin, L.M., Villegas, R., Moro, E., Argenton, F., et al. (2011). Lef1-dependent Wnt/β-catenin signalling drives the proliferative engine that maintains tissue homeostasis during lateral line development. Development *138*, 3931-3941.

S4. Park, H.C., Kim, C.H., Bae, Y.K., Yeo, S.Y., Kim, S.H., Hong, S.K., Shin, J., Yoo, K.W., Hibi, M., Hirano, T., et al. (2000). Analysis of upstream elements in the HuC promoter leads to the establishment of transgenic zebrafish with fluorescent neurons. Developmental Biology *227*, 279-293.

S5. Ahrens, M.B., Orger, M.B., Robson, D.N., Li, J.M., and Keller, P.J. (2013). Whole-brain functional imaging at cellular resolution using light-sheet microscopy. Nature Methods *10*, 413-420.

S6. Wen, L., Wei, W., Gu, W., Huang, P., Ren, X., Zhang, Z., Zhu, Z., Lin, S., and Zhang, B. (2008). Visualization of monoaminergic neurons and neurotoxicity of MPTP in live transgenic zebrafish. Developmental Biology *314*, 84-92.

S7. Aizawa, H., Bianco, I.H., Hamaoka, T., Miyashita, T., Uemura, O., Concha, M.L., Russell, C., Wilson, S.W., and Okamoto, H. (2005). Laterotopic Representation of Left-Right Information onto the Dorso-Ventral Axis of a Zebrafish Midbrain Target Nucleus. Current Biology *15*, 238-243.

S8. Lewis, J.L., Bonner, J., Modrell, M., Ragland, J.W., Moon, R.T., Dorsky, R.I., and Raible, D.W. (2004). Reiterated Wnt signalling during zebrafish neural crest development. Development *131*, 1299-1308.

S9. Gilmour, D.T., Maischein, H.M., and Nüsslein-Volhard, C. (2002). Migration and function of a glial subtype in the vertebrate peripheral nervous system. Neuron *34*, 577-588.

S10. Concha, M.L., Russell, C., Regan, J.C., Tawk, M., Sidi, S., Gilmour, D.T., Kapsimali, M., Sumoy, L., Goldstone, K., Amaya, E., et al. (2003). Local tissue interactions across the dorsal midline of the forebrain establish CNS laterality. Neuron *39*, 423-438.

S11. Talbot, W.S., and Schier, A.F. (1999). Positional cloning of mutated zebrafish genes. Methods Cell Biol. *60*, 259-286.

S12. Dreosti, E., Vendrell Llopis, N., Carl, M., Yaksi, E., and Wilson, S.W. (2014). Left-Right Asymmetry Is Required for the Habenulae to Respond to Both Visual and Olfactory Stimuli. Current Biology *24*, 440-445.
